# Quantitative Proteomic Profiling Identifies DPYSL3 as Pancreatic Ductal Adenocarcinoma-Associated Molecule That Regulates Cell Adhesion and Migration by Stabilization of Focal Adhesion Complex

**DOI:** 10.1371/journal.pone.0079654

**Published:** 2013-12-05

**Authors:** Takeo Kawahara, Naoe Hotta, Yukiko Ozawa, Seiichi Kato, Keiko Kano, Yukihiro Yokoyama, Masato Nagino, Takashi Takahashi, Kiyoshi Yanagisawa

**Affiliations:** 1 Division of Molecular Carcinogenesis, Nagoya University Graduate School of Medicine, Nagoya, Aichi, Japan; 2 Institute for Advanced Research, Nagoya University, Nagoya, Aichi, Japan; 3 Division of Surgical Oncology, Nagoya University Hospital, Nagoya, Aichi, Japan; 4 Department of Pathology and Molecular Diagnostics, Nagoya University Hospital, Nagoya, Aichi, Japan; Schulze Center for Novel Therapeutics, Mayo Clinic, United States of America

## Abstract

Elucidation of how pancreatic cancer cells give rise to distant metastasis is urgently needed in order to provide not only a better understanding of the underlying molecular mechanisms, but also to identify novel targets for greatly improved molecular diagnosis and therapeutic intervention. We employed combined proteomic technologies including mass spectrometry and isobaric tags for relative and absolute quantification peptide tagging to analyze protein profiles of surgically resected human pancreatic ductal adenocarcinoma tissues. We identified a protein, dihydropyrimidinase-like 3, as highly expressed in human pancreatic ductal adenocarcinoma tissues as well as pancreatic cancer cell lines. Characterization of the roles of dihydropyrimidinase-like 3 in relation to cancer cell adhesion and migration *in vitro*, and metastasis *in vivo* was performed using a series of functional analyses, including those employing multiple reaction monitoring proteomic analysis. Furthermore, dihydropyrimidinase-like 3 was found to interact with Ezrin, which has important roles in cell adhesion, motility, and invasion, while that interaction promoted stabilization of an adhesion complex consisting of Ezrin, c-Src, focal adhesion kinase, and Talin1. We also found that exogenous expression of dihydropyrimidinase-like 3 induced activating phosphorylation of Ezrin and c-Src, leading to up-regulation of the signaling pathway. Taken together, the present results indicate successful application of combined proteomic approaches to identify a novel key player, dihydropyrimidinase-like 3, in pancreatic ductal adenocarcinoma tumorigenesis, which may serve as an important biomarker and/or drug target to improve therapeutic strategies.

## Introduction

Pancreatic cancer is the fifth leading cause of cancer death in Japan with more than 24,000 annual deaths [1], while lung cancer is another hard-to-cure cancer with the highest death tolls of more than 70,000 lives a year [2]. Widespread metastasis and/or massive local invasion are commonly present, when they are diagnosed, making long-term survival of these cancers remain unsatisfactory. Thus, it is evident that elucidation of the underlying mechanisms of invasion and distant metastasis is crucial to improve the current dismal outcome. Along this line, we previously established a highly metastatic clone (NCI-H460-LNM35, hereafter referred to as LNM35) of a non-small cell lung cancer cell line, which helped to identify involvement of the *COX-2*, *CLCP-1*, and *DLX-4* genes in cancer metastasis through global expression profiling analysis of LNM35 and its low-metastatic parental clone, NCI-H460-N15 (herein called N15) [3–8]. 

Comprehensive analysis of protein expression patterns in biological materials may improve understanding of the molecular complexities of human diseases, and could be useful to detect diagnostic or predictive protein expression patterns that reflect clinical features. We previously employed Matrix-assisted laser desorption/ionization mass spectrometry (MALDI MS) for expression profiling of proteins in human lung cancer specimens and found that the resultant proteomic patterns could predict various clinical features [9,10]. We have employed quantitative proteomic analysis with the use of a peptide tagging technology, isobaric tags for relative and absolute quantification (iTRAQ), in order to obtain mechanistic insight into metastasis in human lung cancer [11]. However, only limited number of studies in the area of pancreatic cancer research have exploited this high-throughput method, and various proteins thus far identified as differentially expressed during the development of pancreatic cancer have not been studied in detail in order to gain molecular insight into the aggressive nature of this devastating cancer with frequent massive invasion and distant metastasis [12,13]. 

In the present study, we searched for proteins differentially expressed between cancerous and normal pancreatic duct epithelium through proteomic profiling with iTRAQ, which resulted in the identification of high expression of dihydropyrimidinase-like 3 (DPYSL3) in human pancreatic cancer. We also report detailed functional characterizations of DPYSL3 in relation to cancer cell proliferation, invasion, and metastasis by applying a combined proteomic approach with the aid of multiple reaction monitoring (MRM) technology.

## Results

### Identification of differentially expressed DPYSL3 in pancreatic ductal adenocarcinoma

**Figure 1:**
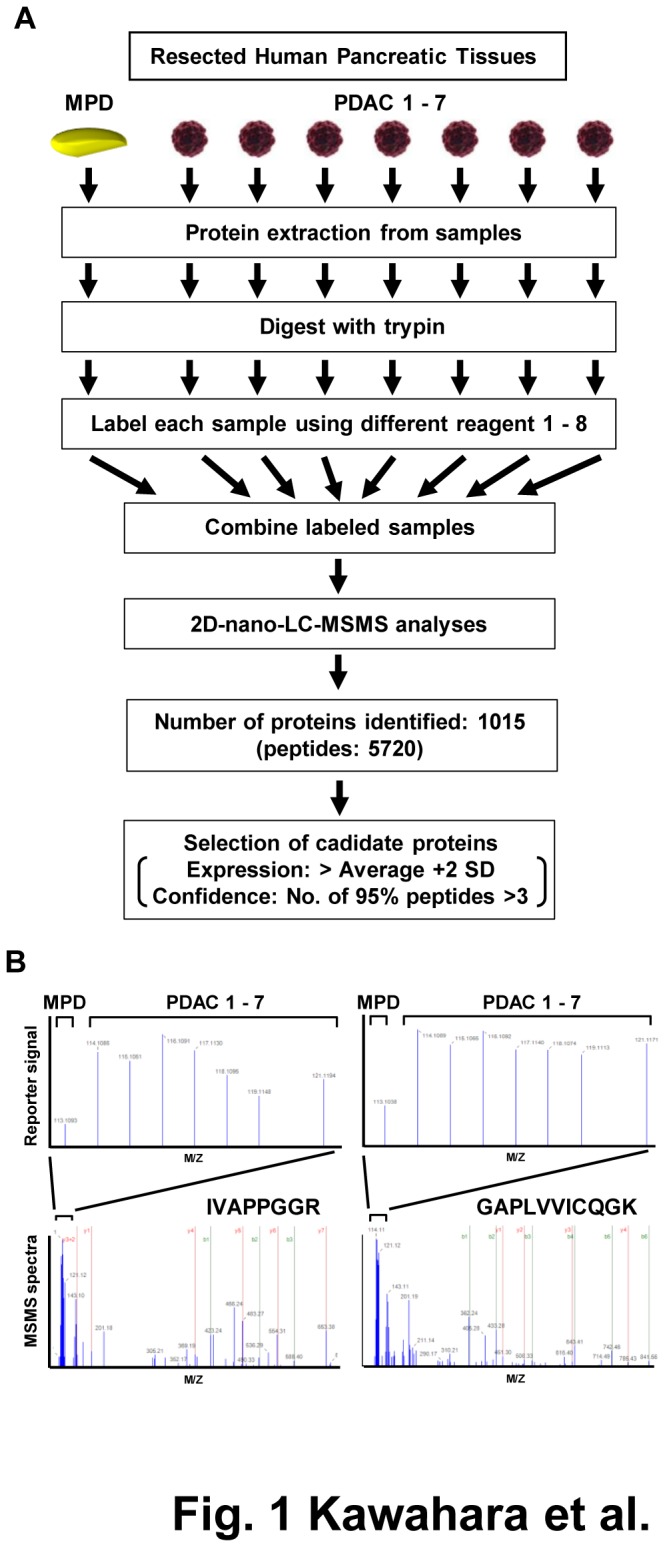
Identification of differentially expressed DPYSL3 in pancreatic ductal adenocarcinoma. (A) Workflow for identification of proteins differentially expressed between pancreatic ductal adenocarcinoma and main pancreatic ductal tissues. Protein Pilot is a program designed for maximizing information obtained from iTRAQ tagging and MS/MS quantification. (B) Spectra obtained from quantification (upper panel) and MS/MS sequencing (lower panel) of representative peptides from DPYSL3 using the Protein Pilot program. MPD, main pancreatic duct; PDAC, pancreatic ductal adenocarcinoma.

**Figure 2:**
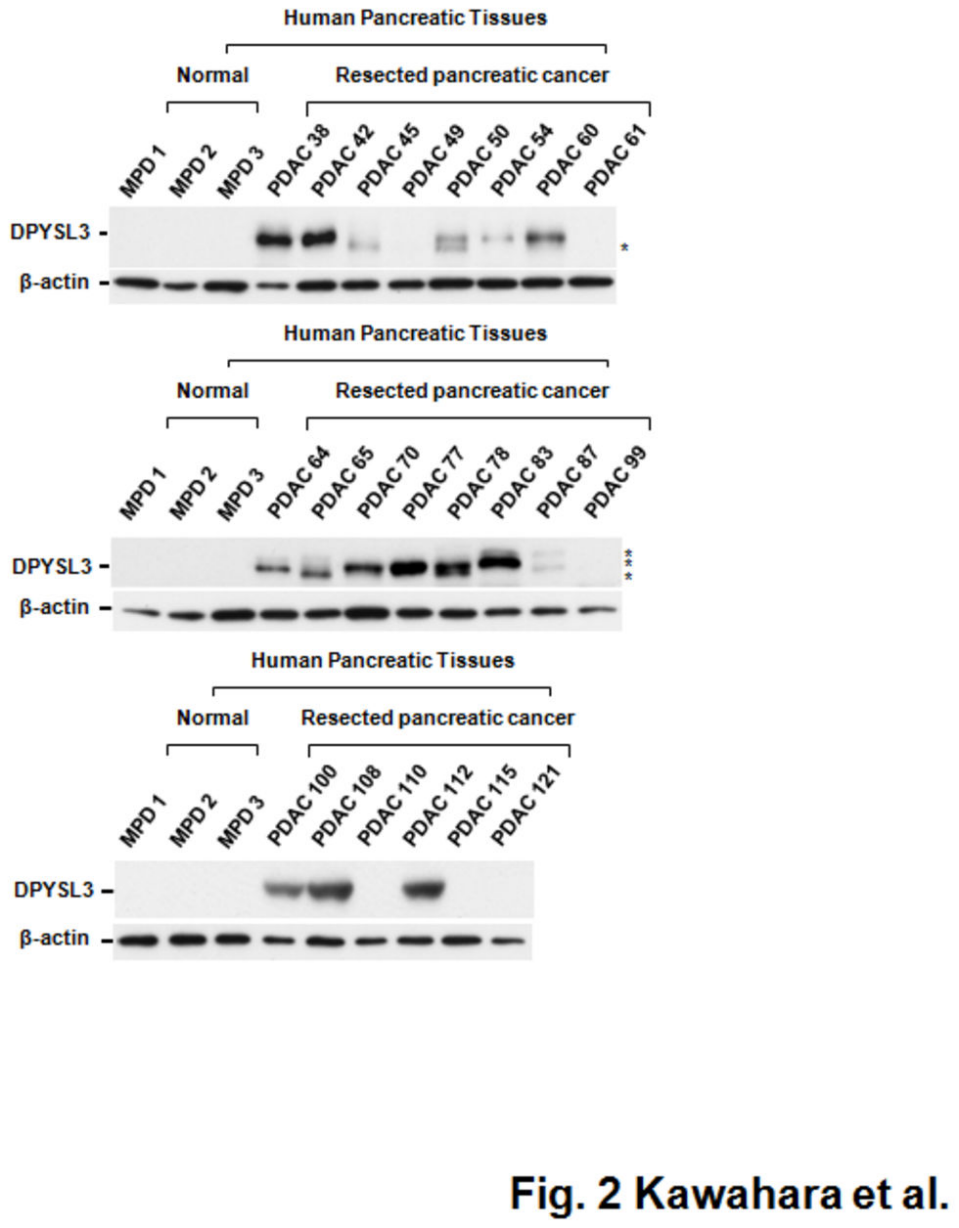
Verification of proteomic data by western blot analyses using independent PDAC tissue specimens. DPYSL3 protein expression in human PDAC tissues not used for the discovery phase with iTRAQ tagging and MS/MS quantification was analyzed by western blotting. MPD, main pancreatic duct; PDAC, pancreatic ductal adenocarcinoma. *Alternative transcriptional variant of DPYSL3.

**Figure 3:**
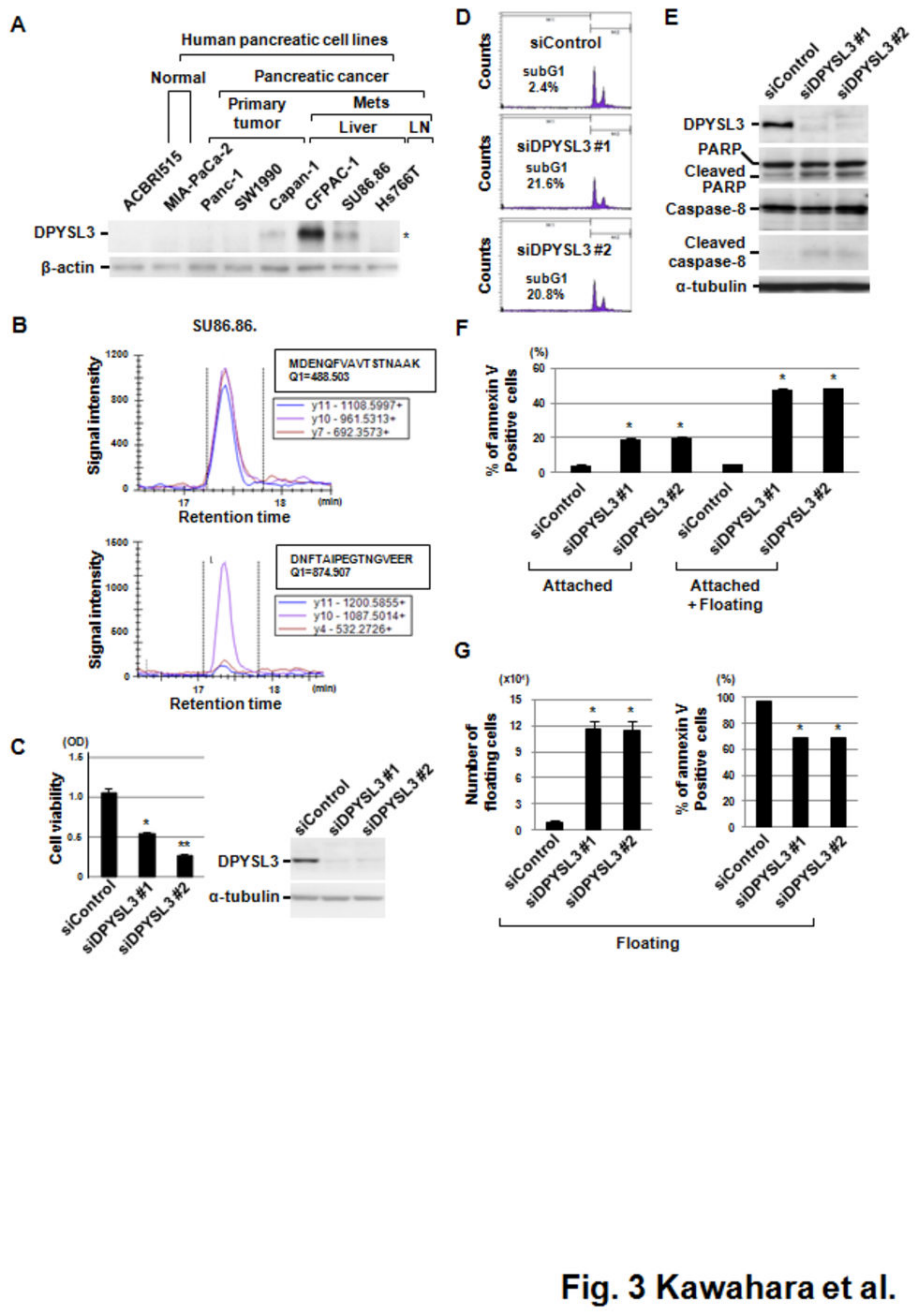
Involvement of DPYSL3 in pancreatic cancer cell survival. (A) DPYSL3 protein expression in human pancreatic cancer cell lines was analyzed by western blotting. β-actin was used as a loading control. (B) MRM analyses using two established transitions revealed that DPYSL3 existed in a SU86.86 cell line. (C) siDPYSL3 treatment induced significant growth inhibition of a CFPAC-1 pancreatic cancer cell line that highly expressed DPYSL3 (left panel). Western blotting showed that DPYSL3 expression was efficiently knocked down by treatment with siDPYSL3. Two different siDPYSL3 proteins (#1 and #2) were used to clarify the specificity of the effect of siRNA-mediated DPYSL3 knockdown. Data are shown as the mean ± SD (n=3). *p <0.002 versus siControl, **p <0.001 versus siControl (Student’s t test). (D) Flow-cytometric analysis showed an increase in the sub-G1 population of CFPAC-1 cells after introduction of siDPYSL3. (E) Western blot analysis showed increased expression of cleaved-PARP and -caspase-8 in siDPYSL3-treated CFPAC-1 cells. (F) Flow-cytometric analysis combined with immunofluorescent staining using annexin V-FITC showed an increase in apoptotic cell death of CFPAC-1 cells after introduction of siDPYSL3. Data are shown as the mean ± SD (n=3). *p <0.001 versus siControl (Student’s t test). (G) siDPYSL3 treatment induced dysfunctions in cellular adhesion and apoptotic cell death. Floating cells were collected from condition media and the number of cells were counted (left panel). Floating cells were also subjected to flow-cytometric analysis combined with immunofluorescent staining using annexin V-FITC showed a significant increase in apoptotic cell death among detached CFPAC-1 cells after introduction of siDPYSL3. Data are shown as the mean ± SD (n=3). *p <0.001 versus siControl (Student’s t test).

We compared the protein profiles between a set of 7 individual fresh-frozen pancreatic ductal adenocarcinoma (PDAC) specimens and a mixture of 3 pooled normal main pancreatic duct (MPD) tissue specimens using mass spectrometry combined with iTRAQ peptide tagging technology, and identified 1015 proteins (Figure 1A and Table S3). For each patient, we selected proteins based on the relative expression in PDAC tissue as compared with pooled MPD that was greater than the average ratio +2 SD, then evaluated the frequency of the selected proteins in the 7 PDAC patients. Accordingly, we found 19 up-regulated proteins that were selected in at least 2 specimens (Table 1). Among those, up-regulation of dihydropyrimidinase-related protein 3 (DPYSL3), histone H2B type 1-J (H2BJ), and glutathione S-transferase P1 (GSTP1) was observed in all 7 of the PDAC specimens (Figure 1B). Up-regulation of H2BJ and GSTP1 is considered to reflect a higher rate of PDAC cell division [15,16] . However, the functional relationship between the characteristics of PDAC and dihydropyrimidinase is unclear. Accordingly, we initially employed western blotting to verify the proteomic data using an independent validation set of PDAC tissue specimens. Expression of DPYSL3 protein was observed in 16 of 22 (77.7%) PDAC tissues, whereas signal of DPYSL3 was not confirmed in the 3 MPD specimens that comprised an independent validation cohort in western blotting (Figure 2 and Table S2). Since mass spectrometric analyses sometimes show higher sensitivity than western blot analyses depending upon specificity and sensitivity of antibody used, though the signal from DPYSL3 were confirmed in 3 pooled MPD in MS profiling, we could not detect it in all of 3 MPD specimens that comprised an independent validation cohort in western blot analyses. Of note, three additional bands other than wild-type DPYSL3 were observed in western blotting analyses, thus we investigated the possibility that these bands reflected modification of DPYSL3. DPYSL3-positive PDAC cell lines (Figure 3A and Figure S1A) were treated with phosphatase and a glycosylation inhibitor and subjected to western blotting assay, however, no significant change was observed (Figure S1B). Since we confirmed DPYSL3 expression in SU86.86 cells, which expresses one of additional products, using MRM analyses with 6 different transitions (Figure 3B and Figure S2), we understand that heterogeneous bands observed in this cell line as well as PDAC tissue specimens may be alternative splicing variants of DPYSL3. We next employed Image J software to compare expression level of DPYSL3 across PDAC patients, and divided them into two groups, high-DPYSL3 and low-DPYSL3, according to DPYSL3/β-actin ratio (Table S2). We conducted statistical analyses to evaluate the significance of DPYSL3 expression in relation to the listed clinical characteristics, however, no significant association between DPYSL3 and clinical characteristics was observed.

**Table 1:** Result from protein expression profiling of patients with pancreatic cancer in the discovery cohort.

**Accession #**	**Name**	**Peptides(95%) **	**PDAC119/3MPD **	**PDAC120/3MPD **	**PDAC122/3MPD **	**PDAC123/3MPD **	**PDAC136/3MPD **	**PDAC138/3MPD **	**PDAC139/3MPD**
IPI00872788	DPYSL3	4	**5.8236**	**4.0091**	**4.8231**	**4.213**	**4.875**	**3.6861**	**3.6631**
IPI00515061	HIST1H2BJ	9	**6.3658**	**5.1424**	**9.2206**	**5.9593**	**8.2686**	**6.5239**	**6.3418**
IPI00219757	GSTP1	5	**5.2444**	**6.9614**	**5.863**	**3.408**	**3.6595**	**7.7206**	**7.011**
IPI00012011	CFL1	4	4.1628	3.1135	3.4594	**4.0643**	**3.3542**	**4.618**	**4.9594**
IPI00552873	HIST1H2AL	5	**4.8203**	**5.241**	3.267	2.2544	3.1102	**5.5493**	**5.2892**
IPI00018146	14-3-3 protein theta	5	1.5423	1.3871	2.2262	**5.2277**	**5.2819**	**3.7495**	**5.7482**
IPI00023006	ACTC1	31	4.2576	**4.3199**	2.4028	**3.1457**	2.7104	**12.315**	**7.8559**
IPI00021263	14-3-3 protein zeta/delta	7	2.642	2.6519	3.3913	**3.1726**	2.8626	**4.0071**	**3.8538**
IPI00647915	TAGLN2	7	4.0373	3.2737	3.0549	2.311	**3.6478**	**4.2864**	**4.0487**
IPI00217465	HIST1H1C	6	2.5444	2.149	**4.4859**	**3.952**	3.019	1.5961	1.829
IPI00645452	TUBB	9	3.3303	**4.1586**	2.3707	2.5416	**4.2104**	3.2809	2.3754
IPI00927101	RPSAP15	3	3.0867	1.7409	3.2011	**3.4315**	**3.9153**	3.1611	2.9445
IPI00553177	SERPINA1	6	2.8568	3.9234	3.3977	**4.8721**	**3.5474**	3.5953	3.3685
IPI00479186	PKM2	23	2.4052	1.9882	1.7363	2.0183	2.3442	**3.9284**	**3.8285**
IPI00027463	S100A6	3	2.0807	3.1992	2.2405	1.0219	1.5851	**4.8025**	**3.9427**
IPI00171611	HIST2H3A	4	1.7747	1.5673	1.2974	1.9209	2.9463	**3.8263**	**3.9559**
IPI00910262	POSTN	8	2.2295	2.0132	1.7284	1.0481	2.9901	**3.8061**	**4.316**
IPI00306959	KRT7	12	1.5053	2.093	2.2755	0.7767	0.9113	**5.0483**	**4.3531**
IPI00013808	ACTN4	18	1.6111	1.828	1.484	1.2117	1.592	**8.6536**	**5.8044**

Value shows relative expression ratio of each protein in PDAC patients compared to MPD. Value written in bold is greater than average + 2SD in protein expression profile of each PDAC specimen.

### Involvement of DPYSL3 in pancreatic cancer cell survival

Next, we examined whether DPYSL3 knockdown affects the viability of pancreatic cancer cells. Since CFPAC-1 cells were shown to highly express DPYSL3 protein and mRNA in western blotting analysis and real-time RT-PCR, respectively, as compared with immortalized normal pancreatic duct cells (ACBRI515) (Figure 3A and Figure S1A), siDPYSL3 was introduced into CFPAC-1 cells and an MTT assay was employed to evaluate the effect of DPYSL3 knockdown. As shown in Figure 3C, cell viability was significantly reduced by addition of siDPYSL3 into DPYSL3-positive CFPAC-1 cells, whereas the DPYSL3-negative MIA PaCa2 and PANC-1 pancreatic cancer cell lines did not show any such effect (Figure S3). Introduction of 2 different siRNAs against DPYSL3 (#1 and #2) showed similar inhibitory effects on cell proliferation, demonstrating the specificity of siRNA-mediated DPYSL3 knockdown (Figure 3C). Flow-cytometric analysis revealed an increase in the sub-G1 population of CFPAC-1 cells after introduction of siDPYSL3, suggesting that the induction of apoptosis is part of the mechanism for reduction of cell viability (Figure 3D). Accordingly, we investigated expressions of cleaved-PARP and -caspase-8 in siDPYSL3-treated CFPAC-1 cells, and found that slight induction of these cleaved products in siDPYSL3-treated adhering cells (Figure 3E). Since siDPYSL3-treated CFPAC-1 cells showed a rounded morphology and many of the cells were detached from the bottom of the culture dishes (Figure S4), we speculated that DPYSL3-knockdown cells die after detachment. To examine this, we employed flow cytometry (FCM) following annexin V-FITC staining. Adhered cells were collected with or without floating cells, then subjected to FCM assays. As shown in Figure 3F, more frequent induction of apoptosis was observed in adhered cells with floating cells treated with siDPYSL3 as compared with the siControl-treated cells. We also analyzed the number of floating cells treated with siControl and siDPYSL3, and found that a much larger number was detached from the bottoms of the dishes after treatment with siDPYSL3 (Figure 3G, left panel). Furthermore, we analyzed the frequency of annexin V-FITC stained cells among floating cells treated with siDPYSL3. As shown in Figure 3G (right panel), 60-70% of the siDPYSL3-treated floating cells were positive for annexin V-FITC staining. These results showed that the floating cells were originally viable and then died from apoptosis.

### DPYSL3 regulates cell adhesion, motility, and invasion in pancreatic cancer cells

**Figure 4:**
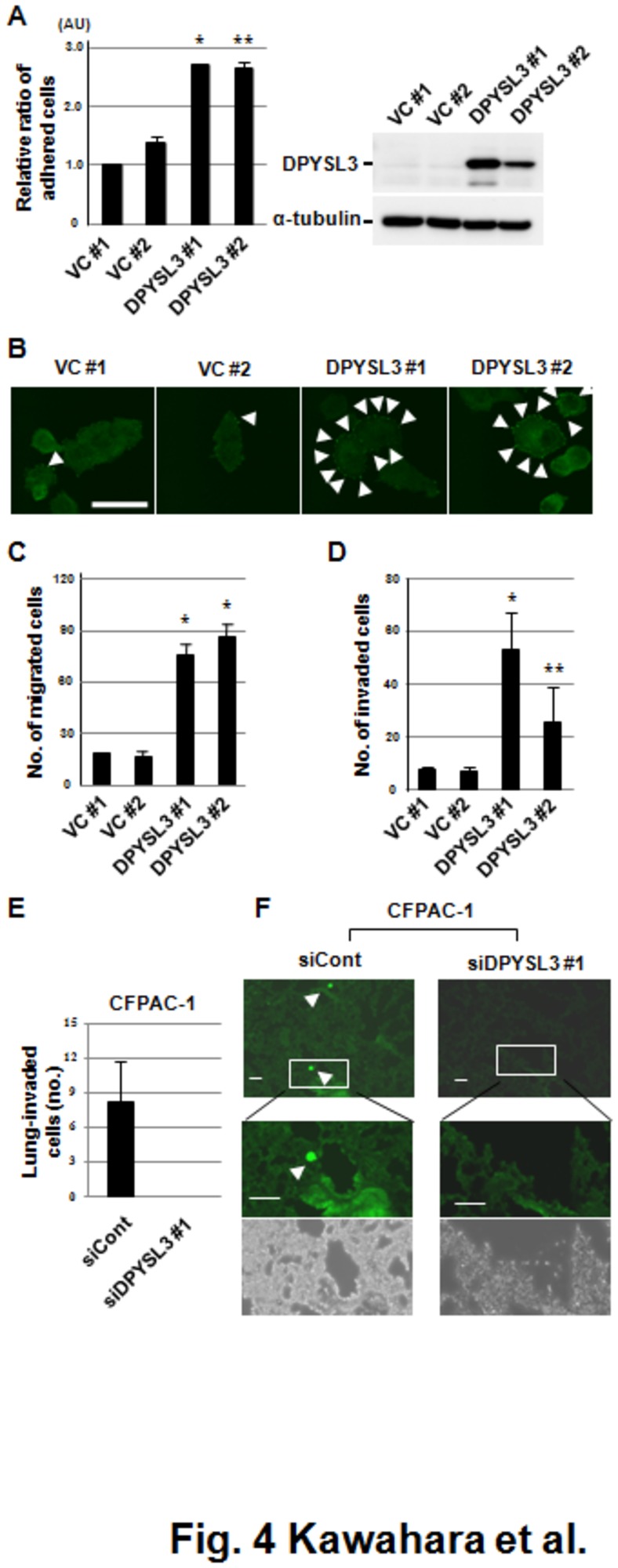
DPYSL3 regulates cell adhesion, motility, and invasion abilities of pancreatic cancer cells. (A) Overexpression of DPYSL3 in PANC-1 cells, which initially showed no expression of DPYSL3, increased the number of cells to adhered fibronectin. Data are shown as the mean ± SD (n=3). DPYSL3 #1 and #2, stable transfectants of DPYSL3 in PANC-1cells; VC #1 and #2, stable transfectants of empty vector in PANC-1cells; *p <0.001 vs. average of VC (Student’s t test). (B) Immunofluorescence staining of vinculin in DPYSL3-expressing PANC-1 cells. Scale bar = 10 μm. Exogenous expression of DPYSL3 in Panc-1 cells promoted formation of larger focal adhesions (arrow heads) as compared with the vector control, supporting the notion that DPYSL3 is the regulatory molecule of cellular adhesion. (C and D) Acquisition of a motile phenotype in DPYSL3-expressing PANC-1 cells was clearly demonstrated by results of a motility assay (C) as well as those of a matrigel invasion assay (D). Data are shown as the mean ± SD (n=5). *p <0.001 vs. average of VC, **p <0.016 vs. average of VC (Student’s t test). (E) Experimental metastasis assay of CFPAC-1 cells knocked down for DPYSL3 with siDPYSL3 #1 (five mice per treatment). Five thin slices were obtained from each mouse lung specimen and images (x20) of each slice were obtained. The number of fluorescent-positive cancer cells was counted and determined for each mouse, then the average value for each treatment was calculated. Bars show the mean ± SD. *p <0.001 vs. average of siControl (Student’s t test). (F) Representative fluorescence images of perfusion-resistant cells. Cells were stained with calcein. Magnified fluorescence images (middle panel) and phase contrast micrographs (lower panel) are shown. Scale bars indicate 50 μm.

Since a dysfunction of cellular adhesion was observed in pancreatic cancer cells treated with siDPYSL3, we further examined the role of DPYSL3 in cell adhesion. Exogenous expression of DPYSL3 in PANC-1 cells, which show no endogenous expression of DPYSL3, increased the number of cells adhered to fibronectin (Figure 4A). This finding prompted us to investigate the characteristics of focal adhesion, which consists of protein complexes including Integrin, focal adhesion kinase (FAK) and adaptor proteins such as Vinculin and Talin1 (TLN1). As shown in Figure 4B, exogenous expression of DPYSL3 in PANC-1 cells promoted formation of larger areas of focal adhesion as compared with the vector control, supporting the notion that DPYSL3 is a regulatory molecule of cellular adhesion. Since focal adhesion is known to regulate cell migration, we next investigated the role of DPYSL3 in pancreatic cancer cell migration and found acquisition of the enhanced motile phenotype in DPYSL3-introduced PANC-1 cells *in vitro*, as shown by a motility assay (Figure 4C) as well as a matrigel invasion assay (Figure 4D). We further evaluated the effects of DPYSL3 on metastasis using a DPYSL3-positive pancreatic cancer cell line, CFPAC-1. CFPAC-1 cells were treated with siDPYSL3 for 24 hours, then injected into the tail vein of mice, which showed significantly reduced experimental lung metastasis at 48 hours after injection (Figure 4E and 4F). We also evaluated the effects of DPYSL3 on metastasis using a DPYSL3-positive highly metastatic lung cancer cell line, NCI-H460-LNM35, which we previously generated through *in vivo* selection [8]. NCI-H460-LMN35 cells were treated with siDPYSL3 for 24 hours, then subjected to a matrigel invasion assay as well as an experimental metastasis assay, which showed significantly reduced the metastatic ability (Figure S5). 

### DPYSL3 interacts with Ezrin and regulates stability of adhesion complex

**Figure 5:**
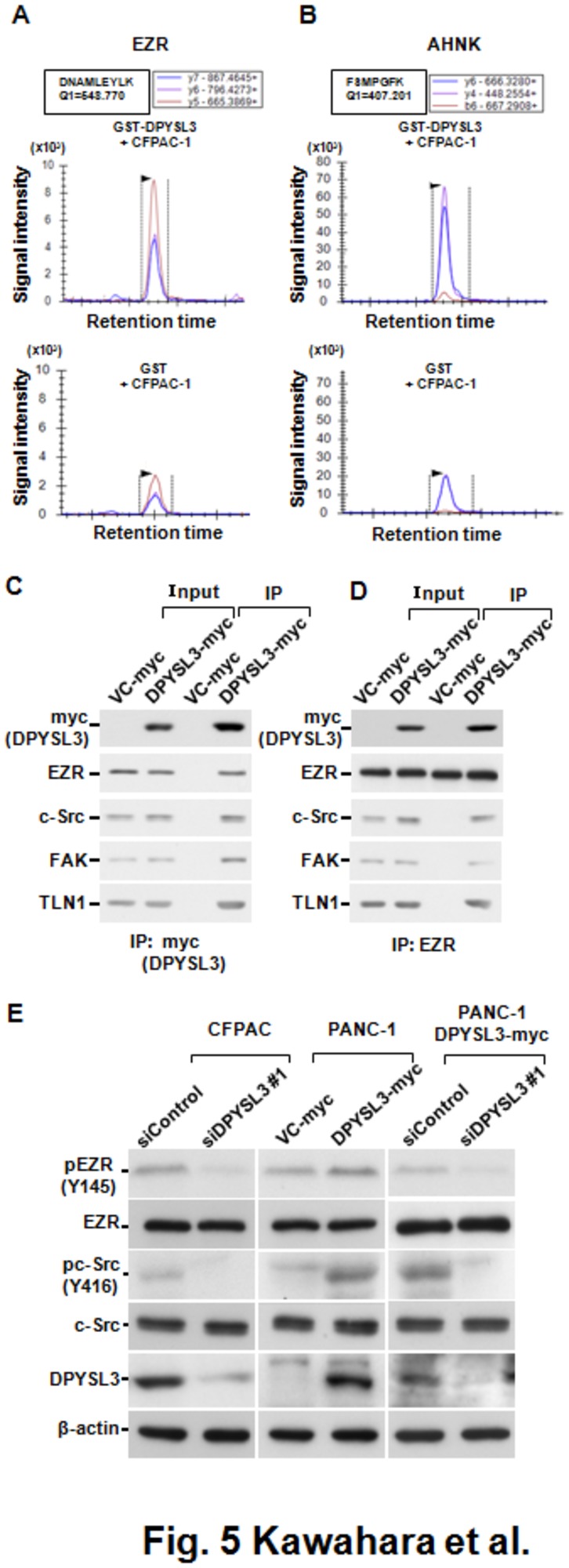
DPYSL3 interacts with EZR and regulates stability of adhesion complex. (A and B) MRM analyses revealed that EZR and AHNK were deteted at higher level in the eluate from the DPYSL3-affinity column to which the CFPAC-1 cell lysate was applied. Eluate samples from the affinity columns were subjected to MRM analysis using an established transition protocol for detection of EZR and AHNK. A clear signal of EZR was observed in that from the DPYSL3-GST-affinity column (A, upper panel). A clear signal of AHNK was observed in that from the DPYSL3-GST-affinity column (B, upper panel). The X-axis shows retention time and y axis signal intensity of the detected products, which reflect the amounts of the peptides of interest. DPYSL3-GST (+), a lysate of Sf9 cells expressing GST-DPYSL3, was applied to glutathione beads; GST, a lysate of Sf9 cells together with purified GST protein, was applied to glutathione beads; CFPAC (+), a lysate of CFPAC-1 cells, was applied to the column. (C) IP-WB analyses using the anti-c-myc antibody for IP in stably DPYSL3-myc transfected PANC-1 cells revealed a clear interaction between DPYSL3 and EZR. FAK, TLN1, and c-Src, constituents of the adhesion complex, were also co-precipitated with DPYSL3. (D) IP-WB analysis using the anti-EZR antibody for IP showed that exogenous expression of DPYSL3 in Panc-1 cells stabilized the interactions between EZR and constituents of the adhesion complex. (E) Western blot analysis showed reduction of phosphorylation of EZR Y145 and c-Src Y416 in CFPAC-1 cells by DPYSL3 knockdown (left panel). Conversely, exogenous expression of DPYSL3 increased the levels of phosphorylated EZR Y145 and c-Src Y416 in PANC-1 cells (middle panel), while those effects were abrogated by simultaneous treatment with siDPYSL3 (right panel).

Our observations revealed that DPYSL3 regulated the adhesion and migration abilities of pancreatic cancer cells *in vitro* as well as metastasis *in vivo*. In order to gain insight into the molecular function of DPYSL3, we employed *in vitro* protein-protein binding assays, followed by comprehensive protein profiling using mass spectrometry (Figure S6A). A total of 17 proteins in the lysate of CFPAC-1 cells were found to be candidate molecules that interact with DPYSL3 (Table S4). To evaluate the significance of their interactions with DPYSL3, we further employed MRM analyses for precise quantification of the concentrations of the proteins of interest in the eluate from the GST-tagged DPYSL3-affinity column as well as from the purified GST-affinity column (Table S4). Consequently, Ezrin (EZR), a cytoplasmic peripheral membrane protein that is known to play a key role in cell surface structure adhesion and migration via activation of FAK and c-Src in the adhesion complex [17–19], was found at higher level in the eluate from the DPYSL3-affinity column, to which the CFPAC-1 cell lysate was applied, than in the eluate from the purified GST-affinity column (Figure 5A and Figure S6B). While a neuroblast differentiation-associated protein (AHNK) (Figure 5B), as well as Vimentin and Lamin-A were also found at higher level in the eluate from the DPYSL3-affinity column, rest of the candidates were not detected in the eluate from the DPYSL3-affinity column (Figure S6C). Since candidate DPYSL3 interacting proteins, identified from proteomic analyses using the GST-tagged DPYSL3 affinity column, may contain non-specific GST tag interacting proteins, we further examined the interaction between DPYSL3 and EZR in PANC-1 cells stably transfected with myc-tagged DPYSL3 using immunoprecipitation-western blot (IP-WB) analysis. As shown in Figure 5C, we confirmed a clear interaction between DPYSL3 and EZR using the anti-myc-tag antibody targeting myc-tagged DPYSL3, which showed that this interaction was specific. Since EZR is crucially involved in formation of an adhesion complex thought to play an important role in development of the metastatic phenotype, we investigated whether DPYSL3 participates in that adhesion complex. We observed that constituents of the adhesion complex, such as FAK, TLN1 and c-Src, were co-immunoprecipitated with DPYSL3 (Figure 5C). Reciprocally, IP-WB analysis using the anti-EZR antibody for IP showed that exogenous expression of DPYSL3 in PANC-1 cells stabilized the interaction between EZR and constituents of the adhesion complex (Figure 5D). Therefore, our results strongly suggest that DPYSL3 is included in the adhesion complex and stabilizes its formation. 

Lastly, we investigated the effects of DPYSL3 expression on tyrosine phosphorylation of EZR (Y145), which is known to be involved in the regulation of cell adhesion, spreading, and proliferation [19]. We consequently observed a reduction of phosphorylation of EZR (Y145) in response to DPYSL3 knockdown in CFPAC-1 cells (Figure 5E, left panel). Conversely, exogenous overexpression of DPYSL3 increased the level of phosphorylated-EZR (Y145) in PANC-1 cells with low DPYSL3 expression (Figure 5E, middle panel). c-Src is a kinase known to be responsible for phosphorylation of the Y145 site on EZR, and phosphorylated EZR (Y145) in turn plays a role in maintenance of activated phosphorylation of c-Src at the Y416 residue [19]. We therefore analyzed the level of phosphorylation of c-Src (Y416) in siDPYSL3-treated CFPAC-1 cells as well as in stable transfectants of PANC-1 cells expressing exogenous DPYSL3. As shown in Figure 5E (left panel), DPYSL3 knockdown resulted in reduced phosphorylation of c-Src (Y416) in CFPAC-1 cells, while exogenous expression of DPYSL3 upregulated the level of phosphorylation of c-Src (Y416) in PANC-1 cells (Figure 5D, middle panel). For further confirmation, we treated PANC-1 cells stably expressing exogenous DPYSL3 with siDPYSL3 and observed that induction of phosphorylated-c-Src (Y416) was cancelled by knockdown of DPYSL3 (Figure 5E, right panel). Taken together, the present findings indicate that DPYSL3 highly expressed in PDAC interacts with EZR and promotes phosphorylations of EZR and c-Src via interaction with EZR as well as with constituents of the adhesion complex. Furthermore, this stabilization of the adhesion complex confers increased adhesion, motility, and invasion abilities to pancreatic cancer cells, which characteristically exhibit highly metastatic phenotypes.

## Discussion

A major challenge in the management of PDAC patients is the nearly inevitable occurrence of tumor metastasis, even in those who are considered to have undergone successful surgical resection. Tumor cells coordinate increased expression levels of metastasis-related genes, which promote cell adhesion, motility, and invasion [20]. The advent of mass spectrometry-based proteomic profiling, in which the expressions of hundreds of proteins can be simultaneously assessed, has greatly facilitated dissection of this process for better understanding of the pathophysiology of PDAC metastasis [11,21]. In the present study, we employed proteomic technologies combining mass spectrometry and peptide tagging to identify a set of proteins whose expression is associated with PDAC tumorigenesis, and identified DPYSL3 as a novel candidate protein. We further demonstrated that DPYSL3 regulates cancer cell migration and adhesion *in vitro* as well as metastasis *in vivo*, and plays an important role in formation of the adhesion complex. Although we clearly showed that functional significance of DPYSL3 in PDAC cells, it is possible that the expression of DPYSL3 occurs in tumor stroma cells or connective tissue with some functional relevance in tumor biology.

DPYSL3 is a member of the DPYSL family of cytosolic phosphoproteins, which mediates semaphorin/collapsin-induced growth cone collapse, and are also involved in axonal guidance and neuronal differentiation [22–24]. During the previous decade, 5 members of the DPYSL gene family (DPYSL1 to 5) encoding closely related 60-66 kDa proteins were isolated [25,26]. However, little is known about the functional significance of the DPYSL family in human malignancies. A previous study reported that DPYSL1 is an invasion suppressor and correlates with clinical outcomes in non-small-cell lung cancer, though the underlying molecular mechanisms of how DPYSL1 regulates the process of cancer metastasis have not been elucidated in detail [27,28]. Interestingly, we found that all three DPYSL3 positive PDAC cell lines were originated from liver metastasis (Capan-1, CFPAC-1 and SU86.86) in the western blotting assays, while Hs766T, originated from lymph node metastasis, does not express DPYSL3. On the other hand the expression of DPYSL3 was not detected in three cell lines originated from primary tumor (MIA PaCa-2, PANC-1 and SW 1990). These results are considered to support the notion that DPYSL3 plays a role in PDAC cell metastasis. In addition, DPYSL3 at 5q32 was recently found to correspond to 1 of 3 additional lung cancer susceptibility loci in a genome-wide association study [29], suggesting the possibility that DPYSL3 may be involved in development of lung cancer. It should also be noted that the molecular function of DPYSL3 in connection with cellular adhesion or stabilization of the adhesion complex has yet to be reported.

EZR is a cytoplasmic peripheral membrane protein that functions as a substrate of protein-tyrosine kinases, and also plays key roles in cellular adhesion and migration in the adhesion complex via activation of FAK and c-Src [17,18]. Phosphorylation is a crucial mechanism that regulates the function of EZR, with c-Src known to phosphorylate the Y145 site on EZR, while phosphorylated EZR (Y145) in turn maintains the activating phosphorylation of c-Src at Y416 residue, making cells active for adhesion, spreading, and proliferation [19]. In the present study, we found that the levels of phosphorylation of EZR and c-Src are regulated by DPYSL3 in pancreatic cancer cells. In addition, the interaction between DPYSL3 and EZR was shown to play a role in stabilization of the adhesion complex that includes c-Src, FAK, and TLN1. Our findings suggest the possible existence of interplay among components of the focal adhesion complex, in which DPYSL3 is crucially involved, and regulates the adhesion and migration of pancreatic cancer cells. It is also of note that DPYSL3 contains a number of consensus phosphorylation sites that may serve as substrates for signaling molecules, such as Cdk, protein kinase C, and proline-directed kinases [23]. Therefore, it would be interesting to elucidate the detailed signaling pathway(s) leading to phosphorylation of DPYSL3 as well as the functional significance of that phosphorylation in the adhesion and migration processes of pancreatic cancer cells.

In the present study, we have shown that the expression of DPYSL3 protein is up-regulated in PDAC using combined proteomic approaches. Given that it has important roles in regulation of motile phenotype of pancreatic cancer cells, DPYSL3 may be an excellent candidate for anti-metastasis therapeutic strategies, which may ultimately lead to a reduction in the large number of deaths caused by this devastating disease.

## Methods

### Cell lines, pancreatic cancer tissues, protein extraction

MIA PaCa-2, PANC-1, SW 1990, CFPAC-1, SU86.86 and Hs766T (human pancreatic cancer cell lines) were purchased from ATCC and maintained in DMEM/10% fetal bovine serum (FBS). Capan-1 (human pancreatic cancer cells) was purchased from ATCC and cultured in IMDM/20% FBS, while ACBRI515 (human pancreatic epithelial cells) was purchased from Cell Systems (Kirkland, WA) and maintained in CS-C medium, which was changed to DMEM/10% FBS 48 hours before analysis. ATCC provided molecular authentication in support of their cell lines.

Tumor and normal main pancreatic ductal tissues (MPD) were collected from patients histologically diagnosed with primary pancreatic ductal adenocarcinoma (PDAC), and who underwent potentially curative resection at Nagoya University Hospital between February 2005 and October 2006. Staging was determined after pathologic evaluation of resected specimens according to the International System for Staging Pancreatic Cancer (Table S1 and S2). All tissues were quickly frozen in liquid nitrogen and stored at -80°C until analysis. All specimens were processed in the same manner. Similarly to the method we usually take for transcriptome analysis [3,5] , protein was isolated from frozen tissues of the tumor and normal MPD specimens, which were subjected to gross microdissection using 10-μm sections cut from frozen tissue samples on a cryostat at –20°C under the guidance of Giemsa staining to reduce contamination of the fibrotic areas and connective tissues using T-PER reagent (Pierce, Rockford, IL), according to the manufacturer’s instructions. Quantities were checked using a Lowry assay (BioRad, Hercules, CA).

### Ethics Statement

Approval from Nagoya University Hospital institutional review board and written informed consent from each patient were obtained. All animal experiments were approved by the Committee on the Ethics of Animal Experiments, Nagoya University Graduate School of Medicine, Japan.

### Mass spectrometry analysis of iTRAQ discovery analyses

An overview of the workflow for the discovery phase is shown in Figure 1A. For iTRAQ labeling, 100 µg of protein extracted from the human resected tissues or cells were reduced, alkylated, and digested with trypsin according to the manufacturer’s instructions (Applied Biosystems Inc., Foster city, CA). For discovery analyses, digested protein prepared from each sample was labeled with the iTRAQ reagent, then labeled samples were pooled and washed according to the manufacturer’s instructions (Applied Biosystems Inc.).

Two-dimensional peptide fractionation was performed with a DiNa Direct Nano-flow LC system (KYA Technologies, Tokyo, Japan) using a strong cation exchange (SCX) column [HiQ sil SCX, 0.5-mm inside diameter (i.d.) x 35 mm], a reverse-phase (RP) trap column (HiQ Sil C18-3, 0.8-mm i.d. x 3 mm), and an RP analytical column (HiQ Sil C18-3 Gradient, 0.15-mm i.d. x 50 mm). Peptides trapped on the SCX column were eluted by injection of ammonium-formate (AF) buffer (pH 3.0, containing 2% acetonitrile) at various concentrations (10, 30, 50, 80, 100, 150, 200, 300, 500 mM). The eluate from each injection of AF buffer was directly subjected to the trap column and sequentially to the analytical column using a gradient of 0-50% solvent B in solvent A over a period of 125 minutes [solvent A: 0.1% formic acid (FA), 2% acetonitrile; solvent D: 0.1% FA, 70% acetonitrile), then 50-100% solvent B for 10 minutes at a flow rate 200 nl/minute. The RP column eluate was analyzed using a Q-STAR ELITE mass spectrometer (Applied Biosystems Inc.) in information-dependent acquisition (IDA) mode with the scan cycles set to perform a 1-second MS scan followed by 3 MS/MS scans for 2 seconds each. The acquisition method was set to allow 1 repetition at any m/z, followed by dynamic exclusion for a period of 60 seconds. Relative protein abundance was determined using the results of MS/MS scans of the iTRAQ-labeled peptides. iTRAQ-labeled peptides were fragmented under collision-induced dissociation (CID) conditions to give fragment ions the sequence information for the peptide and reporter ions. Thus, the identity of the protein from which the peptide was analyzed was confirmed and the ratios of the peak areas of the iTRAQ reporter ions used to compare the relative abundance of the protein identified in the sample.

The software packages used for data acquisition and analysis were Analyst 1.1 and Protein Pilot 4.0, respectively. We searched the Ref-Seq human database provided by NCBI. The confidence score, based on a Protein Pilot generated value, was used to evaluate the quality of the sequence of the identified peptide. For each patient, we selected proteins based on the relative expression in PDAC tissue as compared with pooled MPD that was greater than the average ratio +2 SD, then evaluated the frequency of the selected proteins in the 7 PDAC patients.

### Antibodies

Anti-DPYSL3 was purchased from Millipore (Billerica, MA), pY397-FAK from Abcam (Cambridge, MA), anti-PARP, anti-caspase-8, and anti-Erk from Cell Signaling Technology (Beverly, MA), anti-Lamin and anti-c-myc from Santa Cruz Biotechnology (Santa Cruz, CA), and anti-α-tubulin, anti-β-actin, anti-vinculin, and agarose-conjugated anti-c-myc from Sigma-Aldrich (St Louis, MO).

### Plasmid construction and transfection, and establishment of stable clones

The full length DPYSL3 open reading frame (IMAGE clone 6177053) was obtained from Invitrogen (Carlsberg, CA) and a 4710-bp EcoRI fragment containing the entire coding sequence of DPYSL3 was cloned into pcDNA3 (pcDNA3-DPYSL3). For construction of myc-tagged DPYSL3, a 2052-bp open reading frame was amplified using myc-tag containing a primer and the resultant product was cloned into pcDNA3 (pcDNA3-myc-DPYSL3). Sequence confirmation was conducted thoroughly. PANC-1 cells were transfected with pcDNA3-DPYSL3 or pcDNA3 plasmid using Fugene 6 reagent (Roche, Alameda, CA), according to the manufacturer’s instructions, and selected with the aid of 600 µg/ml of neomycin for 2 weeks to establish stable clones (PANC-1-DPYSL3 #1 and #2, and Panc-1-VC #1 and #2). PANC-1 cells were also transfected with a pcDNA3-DPYSL3-myc or pcDNA3-myc plasmid using Fugene 6 reagent, then selected with the aid of 600 µg/ml of neomycin for 2 weeks to establish stable clones (PANC-1-DPYSL3-myc, PANC-1-VC-myc). The expression of DPYSL3 was confirmed based on western blotting findings.

### Western blot analysis, colorimetric analysis, and flow cytometric analysis

At 24 hours before transfection of siRNAs, 1×10^5^ of CFPAC-1 cells were plated. Knockdown of DPYSL3 was carried out by transfection of 20 nM of siRNA targeting DPYSL3 (two Mission siRNAs: SASI_Hs01_00065697 and SASI_Hs01_00065698, Sigma-Aldrich, St Louis, MO) using RNAi-MAX (Invitrogen), according to the manufacturer’s instructions, with culture media replaced at 24 hours after transfection. Cells were harvested with SDS-sample buffer at 96 hours after transfection and subjected to western blot analysis, and the numbers of viable cells were determined with TetraColor One (Seikagaku, Tokyo, Japan) at 48 hours after transfection. Lysates from CFPAC-1 and SU86.86 cells were treated with 0.2 unit/µl of alkaline phosphatase (TAKARA BIO Inc., Shiga, Japan) for 30 minutes at 37°C. CFPAC-1 and SU86.86 cells were treated with 0.1µg/ml of tunicamycin (glycosylation inhibitor, SIGMA-Aldrich) for 8 hours and then harvested with SDS-sample buffer. Total cell lysates (10 µg) were separated using SDS-PAGE and transferred to membranes. Western blotting results were quantitated using Image J software (http://rsb.info.nih.gov/ij/index.html), according to the instructions. The ratio of expressions of DPYSL3 and β-actin was calculated, then used for comparison of the expression level of DPYSL3 in PDAC patients. Those patients with a ratio higher than average were determined to be high expressers. For flow cytometric analysis, transfected cells were harvested using 0.5% NP-40 at 48 hours after transfection, then cell nuclei were stained with propidium iodide (Sigma-Aldrich) and cellular DNA contents measured using a FACScalibur flow cytometer equipped with the CELLQuest program (BD Biosciences, Bedford, MA). Analyses of induction of apoptosis were conducted using an annexin V-FITC staining kit (BD Biosciences). Attached and/or floating cells were collected at 48 hours after transfection, and stained with annexin V-FITC, following the manufacturer’s instructions. At least 3 independent experiments using colorimetric and flow cytometric assays were performed.

### Adhesion assay

Cells were serum-starved overnight, then resuspended in serum-free DMEM/0.1% BSA and incubated in suspension for 3 hours at 37°C. Six-well plates were coated with 10 μg/ml fibronectin/PBS overnight at room temperature, then washed twice and dried. Next, 3×10^4^ of Panc-1-DPYSL3 or -VC cells were seeded onto the plates and incubated for 1 hour at 37°C in a CO_2_ incubator. The plates were shaken for 60 seconds at 60 rpm twice, and floating cells were removed using 2 wash cycles with PBS. Adherent cells were collected and counted using a Coulter Counter, or lysed with SDS-sample buffer for western blot analysis. Three independent experiments were performed in triplicate.

### Quantitative real-time RT-PCR

Total RNA samples were prepared from MIA PaCa-2, PANC-1, SW 1990, Capan-1, CFPAC-1, SU86.86, Hs766T and ACBRI515 using an RNeasy Mini Kit (QIAGEN, Valencia, CA). Complementary DNA samples were prepared using a High-Capacity cDNA Reverse Transcription Kit (Applied Biosystems Inc.). A TaqMan PCR assay was also performed to quantify DPYSL3 mRNA expression using commercially available FAM^TM^-labeled probes for DPYSL3 and VIC^TM^-labeled probes for 18S, according to the manufacturer’s instructions. Ct values for DPYSL3 were normalized to those of 18S (∆Ct). The average ∆∆Ct values were then calculated after normalization to the ∆Ct value obtained at time point 0.

### Immunofluorescence analysis

Panc-1-DPYSL3 or -VC cells (4× 10^4^) were transferred to cover glasses coated with fibronectin (5 μg/ml PBS), and cultured for 12 hours. The cells were fixed in 3.7% formaldehyde for 10 minutes, treated with PBS containing 0.3% Triton X-100 for 5 minutes, and incubated with anti-vinculin antibody for 1 hour at room temperature. Cells were then washed 3 times with PBS, incubated for 1 hour with Alexa 488-conjugated secondary antibody (Molecular Probes, Carlsberg, CA), and analyzed using a fluorescent microscope.

### In-vivo metastasis assay

An experimental metastasis assay following tail vein injection of tumor cells was performed, essentially as described by [14]. CFPAC-1 cells and NCI-H460-LNM35 cells were transfected with either siControl or siDPYSL3 #1 as described above, then the transfectants were labeled with calcein (BD Biosciences) for one hour at 24 hours after siRNA transefection. Labeled cells were collected and counted, and 1.0 x 10^6^ cells in 0.1 ml of PBS were injected into tail veins of 6-week-old female SCID mice. Two days after injection, the mice were euthanized, then 6ml of PBS was injected into the right ventricle for perfusion of the lung microvasculature. The perfused lungs were embedded in OCT (Sakura), sectioned (thickness 10 mm) with a Leica CM3050 (Leica Microsystems), and fixed using Fluoromount. Perfusion-resistant cells were determined by direct counting in the sections using an A1 Rsi confocal microscope. Five thin slices were obtained from each mouse lung specimen and images (x20) of each slice were obtained. The number of fluorescent-positive cancer cells was counted and determined for each mouse, and then the average value for each treatment was calculated.

### Identification of DPYSL3 and DPYSL3-bound proteins in pancreatic cancer cells

GST-tagged DPYSL3 proteins were expressed in Sf9 insect cells using a Gateway system (Invitrogen), according to the manufacturer’s instructions. GST-tagged DPYSL3 proteins were purified using glutathione-sepharose (GE Healthcare, Waukesha, WI), then mixed with a protein extract from CFPAC-1 cells. DPYSL3-bounded proteins were eluted by addition of 2 mM of glutathione, and reduced, alkylated, and digested with trypsin according to the manufacturer’s instructions (Applied Biosystems Inc.). Further peptide sequence analyses were conducted by mass spectrometry (Thermo Fisher Scientific, Waltham, MA). Transitions for MRM analyses were created with MRM pilot v2.0 according to the manufacturer’s instructions (Applied Biosystems Inc.) using peptide information obtained from sequence analyses of candidate DPYSL3-bounded proteins. An MRM run was performed for the predetermined transitions using a 4000 Q TRAP hybrid triple quadrupole/linear ion trap instrument (AB Sciex, Foster City, CA) in MRM mode. Multiple peptides per candidate protein (at least two peptides for each) and three transition peptides were used. Aliquots (up to 10 μg) of freshly prepared test (purified from a mixture of CFPAC-1 cell lysate, GST-DPYSL3, and glutathione-beads) and negative control (purified from a mixture of CFPAC-1 cell lysate, purified GST protein, and glutathione-beads) samples as well as SU86.86 cell lysate were reduced, alkylated, and digested with trypsin according to the manufacturer’s instructions (Applied Biosystems Inc.). Digested peptide samples were injected into a reverse-phase (RP) trap column (HiQ Sil C18-3, 0.8-mm i.d. x 3 mm) and then separated with an RP analytical column (HiQ Sil C18-3 Gradient, 0.15-mm i.d. x 50 mm) using a gradient of 0-50% solvent B in solvent A over a period of 125 minutes [solvent A: 0.1% formic acid (FA), 2% acetonitrile; solvent D: 0.1% FA, 70% acetonitrile) followed by 50-100% solvent B for 10 minutes at a flow rate 200 nl/minute. MRM transitions were acquired at unit resolution in both Q1 and Q3 quadrupoles to maximize specificity. The scan time was maintained at 50 ms for each transition, and the pause time between transition scans was set to 5 ms. Once electrospray MS data were collected, then the peaks were integrated using quantitation procedures in the Analyst software 1.4.2 (Intelli-Quan algorithm). 

### Immunoprecipitation and pulldown assay

After an overnight culture, 6×10^5^ of Panc-1-pcDNA3-DPYSL3-myc or pcDNA3-myc cells were lysed in 1 ml of NP-40 lysis buffer (20 mM Tris-HCl pH 8.0, 100 mM NaCl, 0.5% NP-40, 5 mM EDTA) supplemented with protease inhibitor and centrifuged at 15,000 rpm at 4°C for 30 minutes. Next, 300 μl of supernatant was transferred to new tubes and incubated with the anti-Ezrin antibody overnight at 4°C. Protein G-sepharose beads (GE Healthcare) were added to the solutions and incubated for 2 hours at 4°C. Immunoprecipitates were extensively washed 4 times and the eluted precipitates were resolved using SDS-PAGE. For pull-down precipitation assays, 300 μl of supernatant from the lysate of PANC-1-pcDNA3-DPYSL3-myc or pcDNA3-myc cells was incubated overnight at 4°C with 15 μl of agarose-conjugated anti-c-myc antibody. Immunoprecipitates were extensively washed 4 times and the eluted precipitates were resolved using SDS-PAGE.

## Supporting Information

Figure S1 **Detection of DPYSL3 transcripts by quantitative real-time PCR and analyses of modification of DPYSL3 protein western blot analyses in pancreatic cancer cell lines.** (A) Total RNA samples were extracted from each cells. Complementary DNA samples were prepared using a High-Capacity cDNA Reverse Transcription Kit (Applied Biosystems Inc.) were subjected using TaqMan probe (Applied Biosystems Inc.). Value of ACBRI515, normal pancreatic cell line, set as 1. Error bars denote SD. (B) Lysates from CFPAC-1 and SU86.86 cells were treated with 0.2 unit/µl of alkaline phosphatase for 30 minutes at 37°C. CFPAC-1 and SU86.86 cells were treated with 0.1µg/ml of tunicamycin (glycosylation inhibitor) for 8 hours and then harvested with SDS-sample buffer. Total cell lysates were separated using SDS-PAGE and transferred to membranes.
(TIF)

Figure S2 **DPYSL3 protein expression in SU86.86 pancreatic cancer cell lines.** MRM analyses using four additional transitions revealed that DPYSL3 existed in a SU86.86 cell line. SU86.86 cell lysates were reduced, alkylated, and digested with trypsin and the analyzed by a 4000 Q TRAP hybrid triple quadrupole/linear ion trap instrument.
(TIF)

Figure S3 **siDPYSL3 treatment in DPYSL3 negative pancreatic cancer cell lines, MIA PaCa-2 and PANC-1.** siRNA against DPYSL3 showed no effect on cell viability. Data are shown as the mean ± SD (n=3).
(TIF)

Figure S4 **DPYSL3 knockdown reduces cell adhesion of pancreatic cancer cell line, CFPAC-1.** Phase contrast micrographs of CFPAC-1 pancreatic cancer cell linesobtained 3 days after siRNA transfection. Marked number of cells were detached from bottom of dish. Lower panels are magnified images of those in upper panel. Arrow head, representative floating cells. Arrow, attached cells. Scale bar, 50 µm.
(TIF)

Figure S5 **DPYSL3 expression in NCI-H460-LNM35 cells.** (A) Higher expression of DPYSL3 was observed in highly metastatic NCI-H460-LNM35 compared to low metastatic parental cell line, NCI-H460-N15. (B) Treatment with siDPYSL3 markedly reduced expression of DPYSL3 . (C) Introduction of siDPYSL3 markedly reduced migration ability in NCI-H460-LNM35 cells. Lower panels show magnified images. (D) Experimental metastasis assay of NCI-H460-LNM35 cells knocked down for DPYSL3 with siDPYSL3 #1 (five mice per treatment). Five thin slices were obtained from lung specimen of each mouse and x20 image was obtained from each slice. The number of fluorescent label positive cancer cells were counted and average value was calculated in each mice. Data are shown as the mean ± SD (n=5). *p < 0.001 versus siControl as determined by Student’s t test. (E) Representative fluorescence images of perfusion-resistant cells. Cells were stained with calcein. Bars indicate 20 μm.
(TIF)

Figure S6 **Identification of binding partner of DPYSL3.** (A) Workflow for proteomic identification and confirmation of DPYSL3 binding proteins in pancreatic cancer cell line. (B) MRM confirmation of candidate proteins supposed to interact with DPYSL3 were identified from MSMS analyses. Interaction between DPYSL3 and EZR protein was validated. (C) Significant interactions between DPYSL3 and some of the candidates were not confirmed in the test sample (CFPAC-1 cell lysate and GST-DPYSL3), nor in the control samples (CFPAC-1 cell lysate and purified GST).
(TIF)

Table S1 **Clinicopathologic characteristics of patients with pancreatic cancer in the discovery cohort.**
(DOCX)

Table S2 **Clinicopathologic characteristics of patients with pancreatic cancer in the validation cohort.**
(DOCX)

Table S3 (XLSX)

Table S4 **MRM transitions for confirmation of DPYSL3 interacting proteins.**
(DOCX)
