# Supplementary figures and images for: Quantitative Proteomic Profiling Identifies DPYSL3 as Pancreatic Ductal Adenocarcinoma-Associated Molecule That Regulates Cell Adhesion and Migration by Stabilization of Focal Adhesion Complex
Source: PLoS One. 2013 Dec 5;8(12):e79654. doi: 10.1371/journal.pone.0079654 (PMC3855176; doi:10.1371/journal.pone.0079654)

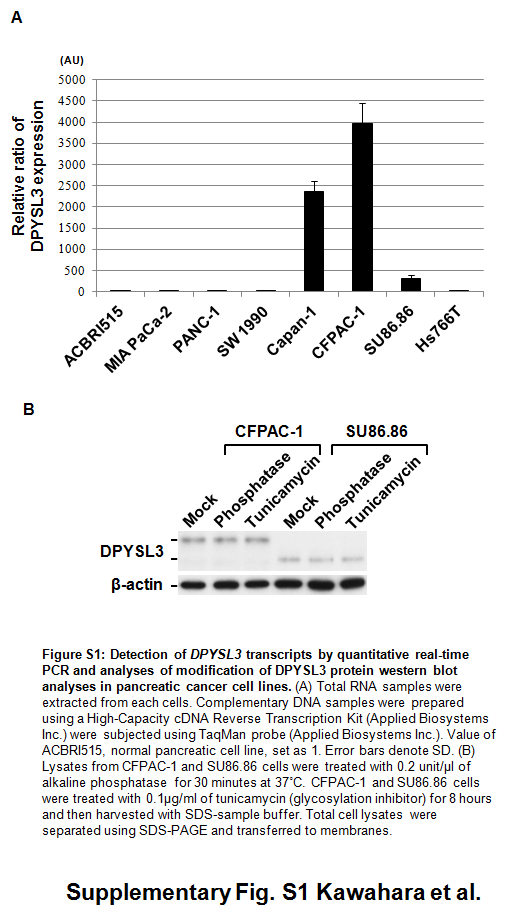

Supplement: Figure S1 — Detection of DPYSL3 transcripts by quantitative real-time PCR and analyses of modification of DPYSL3 protein western blot analyses in pancreatic cancer cell lines. (A) Total RNA samples were extracted from each cells. Complementary DNA samples were prepared using a High-Capacity cDNA Reverse Transcription Kit (Applied Biosystems Inc.) were subjected using TaqMan probe (Applied Biosystems Inc.). Value of ACBRI515, normal pancreatic cell line, set as 1. Error bars denote SD. (B) Lysates from CFPAC-1 and SU86.86 cells were treated with 0.2 unit/µl of alkaline phosphatase for 30 minutes at 37°C. CFPAC-1 and SU86.86 cells were treated with 0.1µg/ml of tunicamycin (glycosylation inhibitor) for 8 hours and then harvested with SDS-sample buffer. Total cell lysates were separated using SDS-PAGE and transferred to membranes. (TIF) [file pone.0079654.s001.tif]

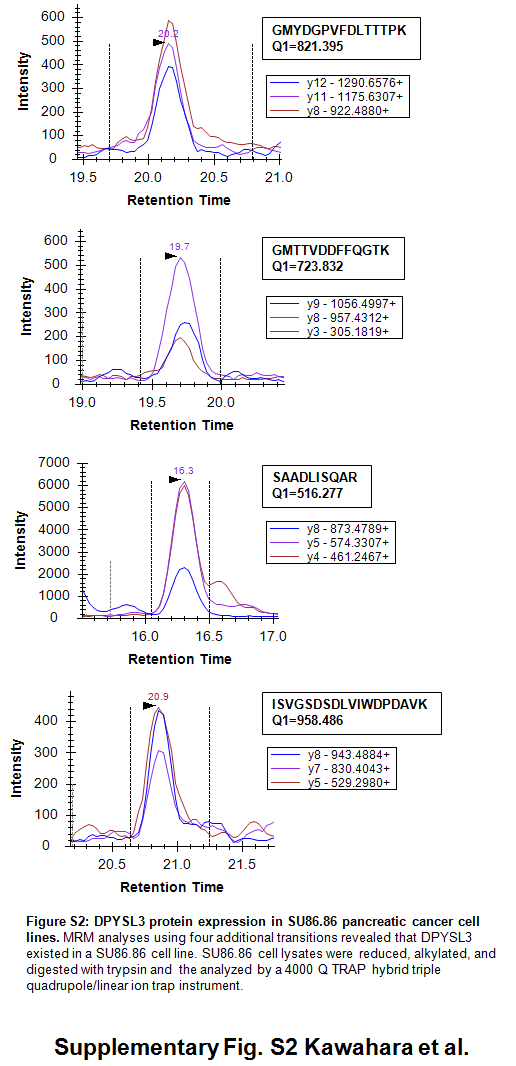

Supplement: Figure S2 — DPYSL3 protein expression in SU86.86 pancreatic cancer cell lines. MRM analyses using four additional transitions revealed that DPYSL3 existed in a SU86.86 cell line. SU86.86 cell lysates were reduced, alkylated, and digested with trypsin and the analyzed by a 4000 Q TRAP hybrid triple quadrupole/linear ion trap instrument. (TIF) [file pone.0079654.s002.tif]

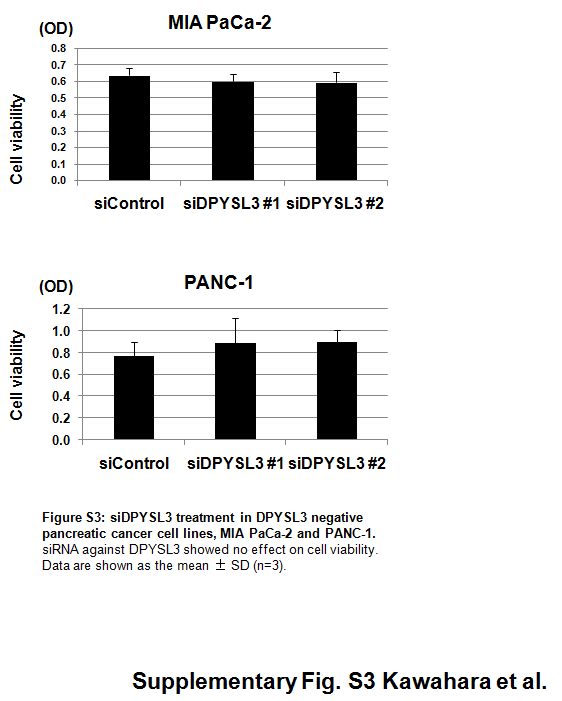

Supplement: Figure S3 — siDPYSL3 treatment in DPYSL3 negative pancreatic cancer cell lines, MIA PaCa-2 and PANC-1. siRNA against DPYSL3 showed no effect on cell viability. Data are shown as the mean ± SD (n=3). (TIF) [file pone.0079654.s003.tif]

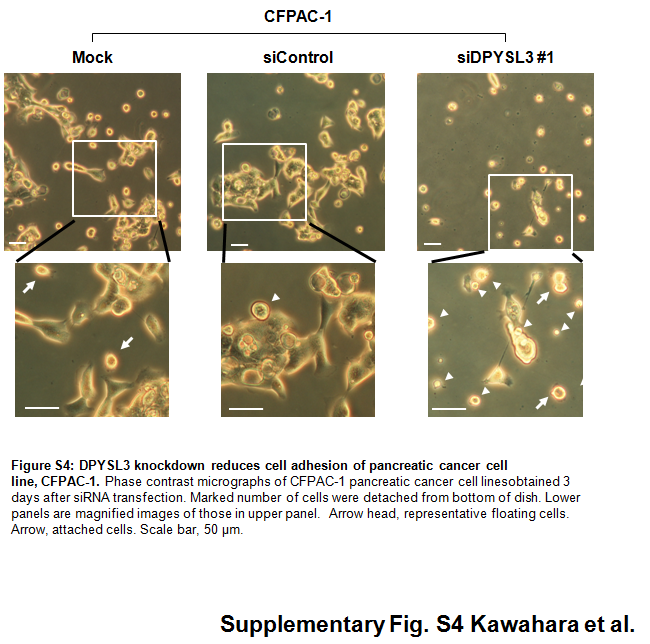

Supplement: Figure S4 — DPYSL3 knockdown reduces cell adhesion of pancreatic cancer cell line, CFPAC-1. Phase contrast micrographs of CFPAC-1 pancreatic cancer cell linesobtained 3 days after siRNA transfection. Marked number of cells were detached from bottom of dish. Lower panels are magnified images of those in upper panel. Arrow head, representative floating cells. Arrow, attached cells. Scale bar, 50 µm. (TIF) [file pone.0079654.s004.tif]

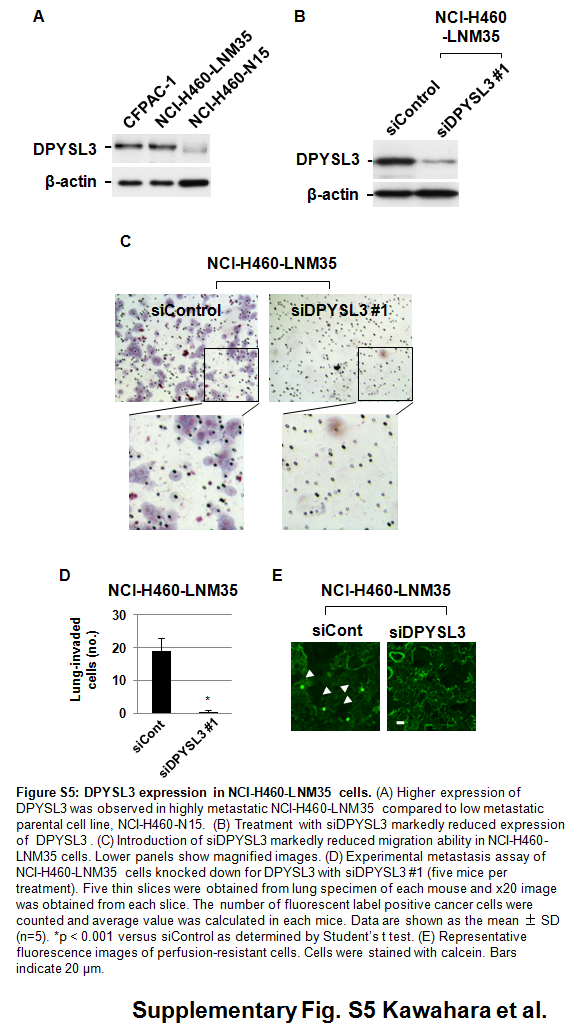

Supplement: Figure S5 — DPYSL3 expression in NCI-H460-LNM35 cells. (A) Higher expression of DPYSL3 was observed in highly metastatic NCI-H460-LNM35 compared to low metastatic parental cell line, NCI-H460-N15. (B) Treatment with siDPYSL3 markedly reduced expression of DPYSL3 . (C) Introduction of siDPYSL3 markedly reduced migration ability in NCI-H460-LNM35 cells. Lower panels show magnified images. (D) Experimental metastasis assay of NCI-H460-LNM35 cells knocked down for DPYSL3 with siDPYSL3 #1 (five mice per treatment). Five thin slices were obtained from lung specimen of each mouse and x20 image was obtained from each slice. The number of fluorescent label positive cancer cells were counted and average value was calculated in each mice. Data are shown as the mean ± SD (n=5). *p < 0.001 versus siControl as determined by Student’s t test. (E) Representative fluorescence images of perfusion-resistant cells. Cells were stained with calcein. Bars indicate 20 μm. (TIF) [file pone.0079654.s005.tif]

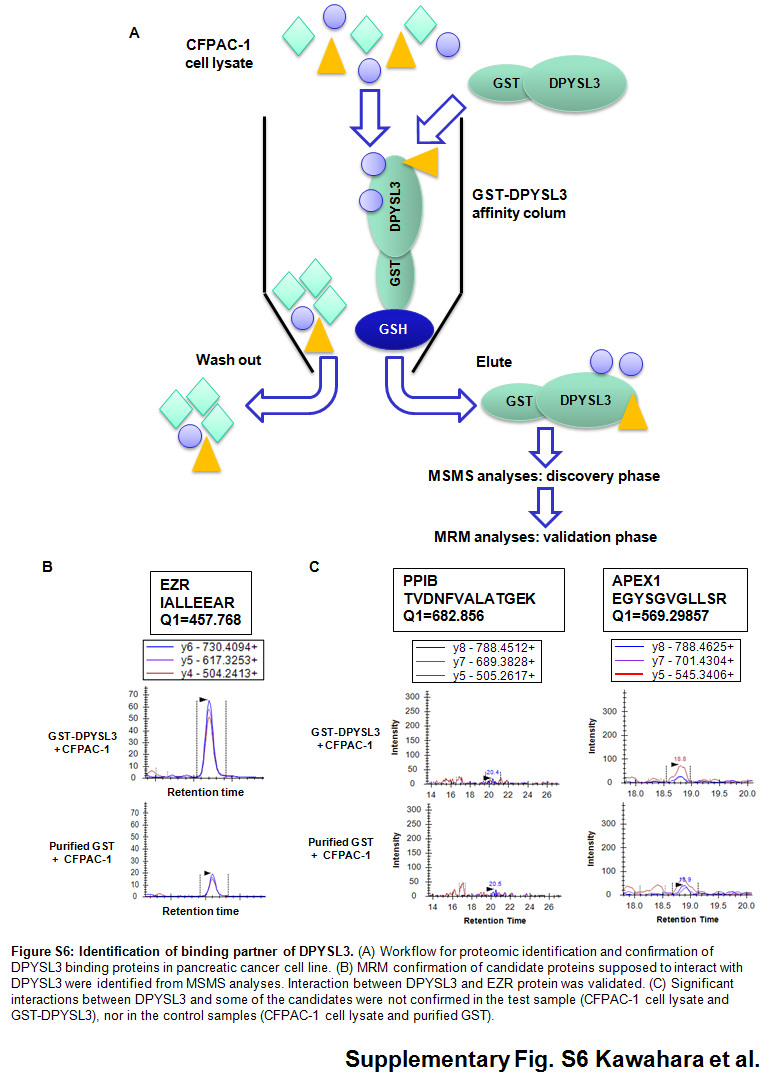

Supplement: Figure S6 — Identification of binding partner of DPYSL3. (A) Workflow for proteomic identification and confirmation of DPYSL3 binding proteins in pancreatic cancer cell line. (B) MRM confirmation of candidate proteins supposed to interact with DPYSL3 were identified from MSMS analyses. Interaction between DPYSL3 and EZR protein was validated. (C) Significant interactions between DPYSL3 and some of the candidates were not confirmed in the test sample (CFPAC-1 cell lysate and GST-DPYSL3), nor in the control samples (CFPAC-1 cell lysate and purified GST). (TIF) [file pone.0079654.s006.tif]
