# Supplementary material for: Quantitative Proteomic Profiling Identifies DPYSL3 as Pancreatic Ductal Adenocarcinoma-Associated Molecule That Regulates Cell Adhesion and Migration by Stabilization of Focal Adhesion Complex
Source: PLoS One. 2013 Dec 5;8(12):e79654. doi: 10.1371/journal.pone.0079654 (PMC3855176; doi:10.1371/journal.pone.0079654)
Supplement: Table S1 — Clinicopathologic characteristics of patients with pancreatic cancer in the discovery cohort. (DOCX) [file pone.0079654.s007.docx]

**Supplementary Table S1. Clinicopathologic characteristics of patients with pancreatic cancer in the discovery cohort**

| Variable | No. of cancer patients in the discovery cohort |
| --- | --- |
| All patients | 7 |
| Age (y.o) | |
| ≤ 70 | 4 |
| > 70 | 3 |
| Sex | |
| Male | 5 |
| Female | 2 |
| pT^#^ status | |
| Tis/1 | 0 |
| T2 | 0 |
| T3 | 4 |
| T4 | 3 |
| pN^#^ status | |
| N0 | 2 |
| N1 | 5 |
| pStage^#^ status | |
| 0 / I | 0 |
| II | 1 |
| III | 3 |
| IVa | 3 |
| IVb | 0 |

^#^pT = pathologic primary tumor; pN = pathologic lymph node status; pStage = pathologic disease stage.
