# Supplementary material for: Quantitative Proteomic Profiling Identifies DPYSL3 as Pancreatic Ductal Adenocarcinoma-Associated Molecule That Regulates Cell Adhesion and Migration by Stabilization of Focal Adhesion Complex
Source: PLoS One. 2013 Dec 5;8(12):e79654. doi: 10.1371/journal.pone.0079654 (PMC3855176; doi:10.1371/journal.pone.0079654)
Supplement: Table S2 — Clinicopathologic characteristics of patients with pancreatic cancer in the validation cohort. (DOCX) [file pone.0079654.s008.docx]

**Supplementary Table S2. Clinicopathologic characteristics of patients with pancreatic cancer in the validation cohort**

| Variable | High expression of DPYSL3 in PDAC pts. | Low expression of DPYSL3 in PDAC pts. | | | P value |
| --- | --- | --- | --- | --- | --- |
| All patients | 8 | 14 | | |  |
| Age (y.o) | | | | | |
| ≤ 70 | 2 | 9 | | | 0.183 |
| > 70 | 6 | 5 | | |  |
| Sex | | | | | |
| Male | 5 | 9 | | 1.000 | |
| Female | 3 | 5 | |  |  |
| pT^#^ status | | | | | |
| Tis/1 | 0 | 0 | | 0.649 | |
| T2 | 0 | 0 | |  |  |
| T3 | 6 | 8 | |  |  |
| T4 | 2 | 6 | |  |  |
| pN^#^ status* | | | | | |
| N0 | 1 | 7 | | 0.167 | |
| N1 | 7 | 7 | |  |  |
| pStage^#^ status | | | | | |
| 0 / I | 0 | 0 | 0.158 | | |
| II | 0 | 2 |  |  |  |
| III | 7 | 6 |  |  |  |
| IVa | 1 | 6 |  |  |  |
| IVb | 0 | 0 |  |  |  |

^#^pT = pathologic primary tumor; pN = pathologic lymph node status; pStage = pathologic disease stage.

*, p = 0.046 as determined by Fisher’s exact test.
