# Supplementary material for: Quantitative Proteomic Profiling Identifies DPYSL3 as Pancreatic Ductal Adenocarcinoma-Associated Molecule That Regulates Cell Adhesion and Migration by Stabilization of Focal Adhesion Complex
Source: PLoS One. 2013 Dec 5;8(12):e79654. doi: 10.1371/journal.pone.0079654 (PMC3855176; doi:10.1371/journal.pone.0079654)
Supplement: Table S4 — MRM transitions for confirmation of DPYSL3 interacting proteins. (DOCX) [file pone.0079654.s010.docx]

**Supplemental Table S4. MRM transitions for confirmation of DPYSL3 interacting proteins.**

| **No.** | **Description** | UniProtKB/Swiss-Prot | Sequence | Q1 | Q3 |
| --- | --- | --- | --- | --- | --- |
| **1** | **Lamin-A** | sp\|P02545\|LMNA_HUMAN | VAVEEVDEEGK | 602.29 | 1033.47 |
|  |  |  |  |  | 934.40 |
|  |  |  |  |  | 805.36 |
|  |  | sp\|P02545\|LMNA_HUMAN | ITESEEVVSR | 574.79 | 1035.50 |
|  |  |  |  |  | 934.45 |
|  |  |  |  |  | 805.41 |
| **2** | **Histone H3.2** | sp\|Q71DI3\|H32_HUMAN | EIAQDFK | 425.72 | 294.18 |
|  |  |  |  |  | 537.27 |
|  |  |  |  |  | 608.30 |
|  |  |  | STELLIR | 416.25 | 401.29 |
|  |  |  |  |  | 514.37 |
|  |  |  |  |  | 643.41 |
| **3** | **Keratin, type II cytoskeletal 8** | sp\|P05787\|K2C8_HUMAN | AQYEDIANR | 540.26 | 473.28 |
|  |  |  |  |  | 717.35 |
|  |  |  |  |  | 880.42 |
|  |  |  | SYTSGPGSR | 456.21 | 473.25 |
|  |  |  |  |  | 560.28 |
|  |  |  |  |  | 661.33 |
| **4** | **DNA-(apurinic or apyrimidinic site) lyase** | sp\|P27695\|APEX1_HUMAN | EGYSGVGLLSR | 569.30 | 545.34 |
|  |  |  |  |  | 701.43 |
|  |  |  |  |  | 788.46 |
|  |  |  | NAGFTPQER | 510.25 | 529.27 |
|  |  |  |  |  | 630.32 |
|  |  |  |  |  | 834.41 |
| **5** | **Histone H2B type 1-C/E/F/G/I ^*^** | sp\|P62807\|H2B1B_HUMAN | ESYSVYVYK | 576.28 | 310.18 |
|  |  |  |  |  | 685.39 |
|  |  |  |  |  | 772.42 |
|  |  |  | LLLPGELAK | 477.31 | 307.68 |
|  |  |  |  |  | 614.35 |
|  |  |  |  |  | 727.43 |
| **6** | **Ezrin** | sp\|P15311\|EZRI_HUMAN | IALLEEAR | 457.77 | 730.41 |
|  |  |  |  |  | 617.33 |
|  |  |  |  |  | 504.24 |
|  |  |  | DNAMLEYLK | 548.77 | 867.46 |
|  |  |  |  |  | 796.43 |
|  |  |  |  |  | 665.39 |
| **7** | **Histone H2B type 1-B ^*^** | sp\|P33778\|H2B1B_HUMAN | ESYSIYVYK | 576.28 | 310.18 |
|  |  |  |  |  | 685.39 |
|  |  |  |  |  | 772.42 |
|  |  |  | LLLPGELAK | 477.31 | 307.68 |
|  |  |  |  |  | 614.35 |
|  |  |  |  |  | 727.43 |
| **8** | **Neuroblast differentiation-associated protein** | sp\|Q09666\|AHNK_HUMAN | ADIDVSGPK | 451.23 | 715.40 |
|  |  |  |  |  | 602.31 |
|  |  |  |  |  | 487.29 |
|  |  |  | FSMPGFK | 407.20 | 666.33 |
|  |  |  |  |  | 448.26 |
|  |  |  |  |  | 667.29 |
| **9** | **Vimentin** | sp\|P08670\|VIME_HUMAN | LGDLYEEEMR | 627.79 | 856.35 |
|  |  |  |  |  | 693.29 |
|  |  |  |  |  | 564.24 |
|  |  |  | ILLAELEQLK | 585.36 | 943.55 |
|  |  |  |  |  | 830.46 |
|  |  |  |  |  | 759.42 |
| **10** | **Histone H4** | sp\|P62805\|H4_HUMAN | DAVTYTEHAK | 567.77 | 585.30 |
|  |  |  |  |  | 748.36 |
|  |  |  |  |  | 849.41 |
|  |  |  | ISGLIYEETR | 590.81 | 697.32 |
|  |  |  |  |  | 810.40 |
|  |  |  |  |  | 1067.54 |
| **11** | **Peptidyl-prolyl cis-trans isomerase B** | sp\|P23284\|PPIB_HUMAN | IEVEKPFAIAK | 415.58 | 201.64 |
|  |  |  |  |  | 501.80 |
|  |  |  |  |  | 646.39 |
|  |  |  | TVDNFVALATGEK | 682.86 | 689.38 |
|  |  |  |  |  | 505.26 |
|  |  |  |  |  | 788.45 |
| **12** | **Histone H3.3 ^**^** | sp\|P84243\|H33_HUMAN | EIAQDFK | 425.72 | 294.18 |
|  |  |  |  |  | 537.27 |
|  |  |  |  |  | 608.30 |
|  |  |  | STELLIR | 416.25 | 401.29 |
|  |  |  |  |  | 514.37 |
|  |  |  |  |  | 643.41 |
| **13** | **Histone H3.1 ^**^** | sp\|P68431\|H31_HUMAN | EIAQDFK | 425.72 | 294.18 |
|  |  |  |  |  | 537.27 |
|  |  |  |  |  | 608.30 |
|  |  |  | STELLIR | 416.25 | 401.29 |
|  |  |  |  |  | 514.37 |
|  |  |  |  |  | 643.41 |
| **14** | **Elongation factor 1-alpha 1** | sp\|P68104\|EF1A1_HUMAN | IGGIGTVPVGR | 513.31 | 428.26 |
|  |  |  |  |  | 685.40 |
|  |  |  |  |  | 912.53 |
|  |  |  | LPLQDVYK | 488.28 | 652.33 |
|  |  |  |  |  | 765.41 |
|  |  |  |  |  | 862.47 |
| **15** | **Core histone macro-H2A.1** | sp\|O75367\|H2AY_HUMAN | AGVIFPVGR | 458.27 | 428.26 |
|  |  |  |  |  | 575.33 |
|  |  |  |  |  | 688.41 |
|  |  |  | SIAFPSIGSGR | 546.30 | 673.36 |
|  |  |  |  |  | 820.43 |
|  |  |  |  |  | 891.47 |
| **16** | **Histone H2A type 1-B/E** | sp\|P04908\|H2A1B_HUMAN | AGLQFPVGR | 472.77 | 428.26 |
|  |  |  |  |  | 575.33 |
|  |  |  |  |  | 703.39 |
|  |  |  | HLQLAIR | 425.77 | 472.32 |
|  |  |  |  |  | 600.38 |
|  |  |  |  |  | 713.47 |
| **17** | **Actin, aortic smooth muscle** | sp\|P62736\|ACTA_HUMAN | AGFAGDDAPR | 488.73 | 343.21 |
|  |  |  |  |  | 630.28 |
|  |  |  |  |  | 701.32 |
|  |  |  | SYELPDGQVITIGNER | 895.95 | 689.36 |
|  |  |  |  |  | 1086.59 |
|  |  |  |  |  | 1298.67 |

*, **: These pairs of proteins are registered as same molecules in UniProt database.
